# Post-Event Application of Neurotropin Protects against Ischemic Insult toward Better Outcomes in a Murine Model of Subarachnoid Hemorrhage

**DOI:** 10.3390/biomedicines9060664

**Published:** 2021-06-10

**Authors:** Tatsushi Mutoh, Shuzo Yamamoto, Takahiro Moriya

**Affiliations:** 1 Department of Aging Research and Geriatric Medicine, Institute of Development, Aging and Cancer, Tohoku University, Aoba-ku, Sendai 980-8575, Japan; shuzo.yamamoto.c7@tohoku.ac.jp; 2 Department of Pharmacology, School of Pharmaceutical Sciences, Ohu University, Koriyama, Fukushima 963-8611, Japan; k-ssaki@gaea.ocn.ne.jp

**Keywords:** early brain injury, neurocognitive function, neurotropin, mouse model, subarachnoid hemorrhage

## Abstract

Early brain injury (EBI) is closely linked to the development of delayed cerebral ischemia and poor outcomes after aneurysmal subarachnoid hemorrhage (SAH). This study aimed to evaluate the neuroprotective effect of neurotropin on EBI in a murine model of SAH. Twenty-four C57BL/6N mice were treated with intraperitoneal injections of either saline or 2.4 units of neurotropin at 1 h after SAH induction and for 3 days consecutively. SAH was created by an endovascular perforation method. In addition to the assessment of cerebral infarction and survival rate, motor and neurocognitive functions were also measured after SAH. Compared to the saline control group, the neurotropin group showed better recovery from locomotive and neurological declines after SAH. The neurotropin group also showed lower rates of post-SAH acute cerebral infarction and better memory and route-learning scores (*p* < 0.05). Meanwhile, there was no significant between-group differences in the overall mortality, hemodynamic parameters, or body weights. In conclusion, post-event treatment with neurotropin could be protective against EBI, lowering the incidence of ischemia and improving some motor and neurocognitive functions after SAH.

## 1. Introduction

Subarachnoid hemorrhage (SAH) is the leading cause of acute death and permanent disability in people with stroke worldwide [1]. Clinical and laboratory investigations have shown that early brain injury (EBI) is closely linked to poor outcomes and the development of delayed cerebral ischemia after SAH [2,3]. However, despite increasing data on the methods and/or drugs for regulating each potential mechanism, the therapeutic options for EBI are still limited, presumably due to its multifactorial causes [4].

Neurotropin is a nonprotein extract derived from the inflamed skin of rabbits inoculated with the vaccinia virus. As it contains multiple physiologically active substances such as amino acids and nucleotides (e.g., ethanolamine, ornithine, uracil, and xanthine) [5], neurotropin is widely used in medical practices for treating chronic pain and allergies in Japan and China [6,7,8]. However, evidence for the neuroprotective effects of neurotropin on the central nervous system is still limited. Two clinical studies have shown that neurotropin is helpful in treating brain edema related to acute ischemic stroke [9,10]. Laboratory investigations have also shown that a 3-week oral intake of neurotropin prior to focal cerebral ischemia leads to reduced cerebral infarction and edema and improved neurological outcomes in a mouse model of temporary ischemic stroke [11]. In the experimental setting, neurotropin upregulated the brain-derived neurotropic factor (BDNF) levels in the cerebral cortex and enhanced the spatial learning, supporting the preferable neuroprotective effects for post-stroke cognitive decline. A more recent study in a murine hypoxic–ischemic brain injury model also showed the potential benefit of neurotropin applied immediately after stroke events. Neurotropin suppressed the brain proinflammatory cytokines and ultimately improved the neurological outcomes [12]. It is known that the antiallodynic effect of neurotropin is mediated by activation of the descending pain inhibitory system (e.g., noradrenergic and serotonergic mechanisms) [13,14]. In addition, animal studies have shown that neurotropin has a direct inhibitory effect on the peripheral nervous system, in which GABAergic interneurons may be involved [15,16]. In a recent in vitro study using immortalized murine hippocampal neurons, neurotropin exhibited a potent neuroprotective effect on inhibiting hippocampal neuronal damage induced by oxidative stress to alleviate amyloid β deposition [17]. However, translational data from animal models are lacking, and thus, a post-SAH therapeutic protocol that included neurotropin has not been established in humans.

Thus, this study aimed to evaluate the “post-event” neuroprotective role of neurotropin in EBI after experimental SAH in mice. We used noninvasive multimodal methods using transcranial/cardiac color Doppler ultrasound and magnetic resonance imaging (MRI) (Figure S1A) that were successfully applied in the same murine model to evaluate the real-time hemodynamic effect of post-SAH EBI in our recent publication [18,19]. We hypothesized that if neurotropin was applied early after SAH, then it could act against EBI and may further provide better outcomes.

## 2. Materials and Methods

### 2.1. Study Design and Animals

This was an in vivo study of male 7-week-old C57BL/6N mice purchased from Japan SLC (Hamamatsu, Japan). The mice were housed in groups of 4 to 5 per cage in an animal facility with a 12 h/12 h light/dark cycle, with food and water available ad libitum. The mice were used around 10 weeks of age.

### 2.2. Experimental SAH Model

SAH was induced via the endovascular perforation technique under isoflurane anesthesia, as previously described [20]. Briefly, a midline skin incision was made in the neck to expose the left carotid bifurcation and external carotid artery (ECA). After ligating the distal part of the ECA, a 5–0 monofilament was gently inserted and advanced into the internal carotid artery through the carotid bifurcation. This was pushed 10–13 mm forward until resistance was felt at the bifurcation of the terminal internal carotid artery and proximal portion of the middle cerebral artery. Then, it was further advanced by 1 to 2 mm to perforate the vessel. Mice were administered warm, sterile isotonic fluids subcutaneously at 3–5% of their body weight prior to and at the end of surgery.

### 2.3. MRI Data Acquisition

Whole-brain MRI was performed using a 1.05-Tesla MR scanner (M7 Compact MRI; Aspect Imaging, Shoham, Israel) under isoflurane anesthesia to ensure the correct head positioning. We obtained T2-weighted images with fast spin echo sequences (field of view (FOV), 22 × 22 mm; slice thickness, 1 mm; repetition time (TR)/echo time (TE), 3000/56.6 ms; matrix size, 128 × 128; resolution, 171 μm; scan time, 14 min); T2*-weighted image (T2*WI) with gradient echo sequences (FOV, 25 × 25 mm; TR/TE, 250/5 ms; flip angle, 45º; matrix size, 128 × 128 × 50; resolution, 195 μm; scan time, 11 min 44 s); and diffusion-weighted imaging (DWI) with fast spin echo sequences (FOV, 25 × 25 mm; slice thickness, 1 mm; TR/TE, 1500/40 ms; matrix size, 128 × 128 (b-value, 1005; large delta, 40 ms; small delta, 10 ms); resolution, 195 μm; scan time, 14 min 24 sec). The severity of SAH was defined on day 1 according to the previously published MRI-based criteria using T2*WI as follows: grade 1 = minimal/localized SAH with no intraventricular hemorrhage (IVH), grade 2 = minimal/localized SAH with IVH, grade 3 = thick/diffuse SAH (hematoma ≥1 mm thick visible in ≥2 slices) with no IVH, and grade 4 = thick/diffuse SAH with IVH (Figure S1B) [20]. For the assessment of newly developed cerebral infarction, hyperintense signals on DWI and T2WI (Figure S1C) were assessed on days 1 and 3.

### 2.4. Hemodynamic Measurements

Ultrasound imaging was performed using the Vevo system (FUJIFILM VisualSonics, Toronto, ON, Canada) heated to 37 °C. The animal’s heart rate (HR) and respiratory rate under isoflurane anesthesia were continuously monitored. The head and chest were shaved, and prewarmed ultrasound gel was applied to the recording areas. Color flow images of the bilateral middle cerebral arteries at the level of the basal ganglia were obtained with a linear array transducer (MX400, FUJIFILM VisualSonics) (Figure S2A) [21,22]. For Doppler echocardiography measurements, the left ventricular outflow tract velocity–time integral (VTI) was calculated by tracing around the waveform at the aortic outflow (Figure S2B) [23,24,25]. The cardiac output (CO) was calculated as the product of stroke volume (calculated from the product of VTI and the cross-sectional area of the aortic valve) and HR. Blood oxygen saturation (SpO_2_) and pulse rate were monitored with a pulse oximeter applied to the paw.

### 2.5. Neurological and Locomotive Function Tests

Functional outcomes were measured daily until post-SAH day 3 using a neurological score and an open-field test. Neurological scores of 3–18 in single-increment steps (higher scores indicate better function) [26] were evaluated by a single, blinded observer. The open field consisted of a square arena (50 × 50 × 50 cm) with which all animals were unfamiliar. Each mouse was placed in the center of the field, and locomotor activity was monitored for 10 min using a CCD video camera and a computerized analysis system (Videotrack; ViewPoint, Lyon, France). In this study, the total distance traveled and the ratio of the central area (within 30 × 30 cm from the center) distance/total traveled distance (an index of exploration ability) were used to assess the recovery of locomotor activity after SAH [21,22]. Rodent memory and route-learning capabilities were evaluated in the subacute to chronic phases at 1 to 3 weeks after SAH using the Y-maze test [27]. Mice were placed in the upper arm of the Y-shaped maze to determine if they could freely move within the 5-min period. Then, the number of visited arms and the alternation index (arm combination)/(total number of visited arms-2) × 100 (%) were calculated.

### 2.6. Experimental Protocols

**Figure 1:**
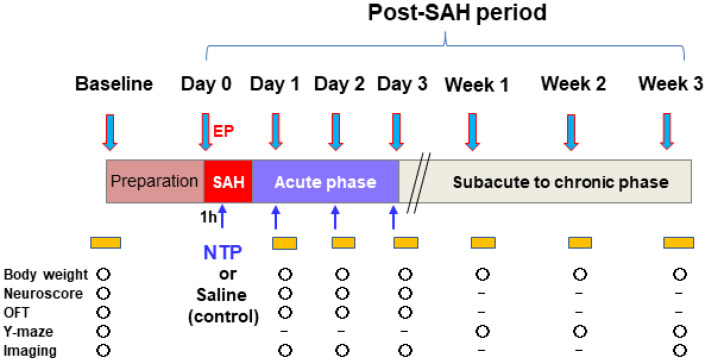
Experimental protocol EP, endovascular perforation, to create SAH. NTP, neurotropin; OFT, open-field test.

Mice that survived the surgical preparation were randomly assigned to two groups. The control group was administered 0.9% physiological saline, whereas the neurotropin group was administered 2.4 units of neurotropin (Nippon Zoki Pharmaceutical Co., Osaka, Japan) injected intraperitoneally at 1 h and once daily up to day 3 after SAH induction. The neurotropin dose was defined based on our pilot experiment and previous data [28]. The primary outcome measures were the occurrence of newly developed cerebral infarction assessed by MRI DWI and T2WI and overall mortality during the 3-week study period. The secondary outcome measures included body weights and functional parameters measured in accordance with the schedule described in Figure 1. All imaging examinations were carried out under 1% isoflurane mixed with 30% O_2_ and 70% medical air at 4 time points: before (baseline) and daily until day 3 after SAH induction. After completion of the final data measurements, the surviving animals were sacrificed under 5% isoflurane anesthesia via a supplemental intraperitoneal injection of sodium pentobarbital (120 mg/kg).

### 2.7. Statistical Analysis

The results were presented as the mean values ± standard deviation (SD) or absolute numbers (%), unless otherwise indicated. When comparing the incidences of acute cerebral infarction and neurological outcomes, we estimated an 80% power to detect a 25% difference between groups, with *n* = 5–8 per group, based on an analysis of variance (ANOVA) model at a significance level of 5%. Continuous data that were normally distributed according to the D’Agostino–Pearson normality test were compared using two-way ANOVA with post hoc Bonferroni–Dunn correction where appropriate. For variables that did not pass the normality test, the Mann–Whitney test (for comparisons between two groups) or Kruskal–Wallis test, followed by the Dunn’s multiple comparison test (for comparisons among three or more independent groups), was performed. Associations of the binary data (newly developed infarction and survival) were tested using Fisher’s extract test. Kaplan-Meier survival analysis and log-rank test were used to analyze mice survival. All statistical analyses were performed using SPSS (version 27; IBM, Chicago, IL, USA) and Prism (version 9: GraphPad Software, San Diego, CA, USA). A *p*-value less than 0.05 was considered statistically significant.

## 3. Results

SAH was successfully induced in 24 mice. In total, 18 (44%) and six (36%) mice had grade 3 and grade 4 SAH, respectively, as assessed during MRI T2*WI (Figure S1B). There were no significant differences in the grade distribution between the control and neurotropin groups (*p* = 0.32). The distributions of the SAH grades were also similar to our previous laboratory studies [20,29] and compatible with the clinical findings [30].

**Figure 2:**
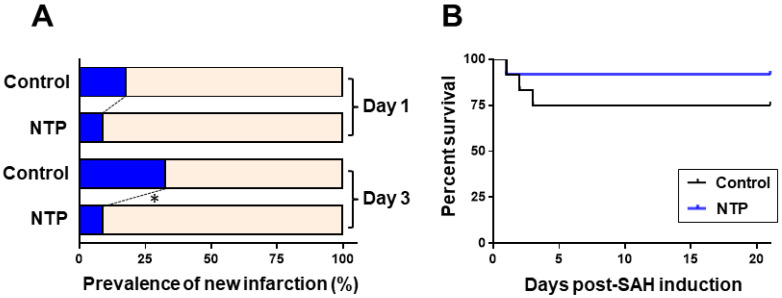
Effects of neurotropin on newly developed cerebral infarction (**A**) and the Kaplan–Meier survival curve (**B**) in mice after-SAH. * *p* < 0.05; Fisher’s extract test. NTP, neurotropin.

Neurotropin showed lower rates of post-SAH new cerebral infarction (*p* = 0.03). DWI-confirmed acute cerebral infarction after SAH (Figure S1C) was detected in two mice (*n* = 1 per each group) on post-SAH day 1 and in seven mice (*n* = 6, control; *n* = 1, neurotropin) on day 3 (Figure 2A). No further development of infarction was detected in the chronic phase. All animal mortalities occurred during the acute phase between day 1 and day 3. The overall mortality rate throughout the study period was 33% (*n* = 3) in the control group and 8% (*n* =1) in the neurotropin group (Figure 2B), and there was no significant between-group difference in the overall mortality (*p* = 0.30).

**Figure 3:**
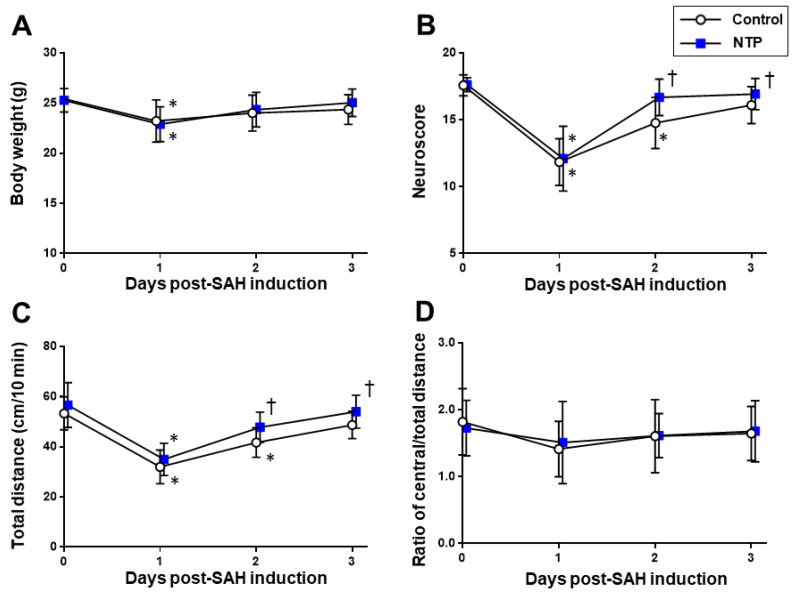
Time–course changes of the cardiorespiratory and cerebrovascular parameters in the acute phase after SAH induction in the control and neurotropin groups. Body weight (**A**). Neuroscore (**B**). Total distance (**C**) and ratio of the central/total distance (**D**) obtained in the open-field test. * *p* < 0.05 vs. baseline; ^†^ *p* < 0.05 vs. control; two-way ANOVA with a post hoc analysis (Bonferroni–Dunn’s test). NTP, neurotropin.

In both groups, significant impairments of the neurobehavioral and locomotive functions, as assessed by the neurological scores and open-field test, were uniformly observed on post-SAH day 1 (*p* > 0.05). All of the parameters were gradually recovered close to the baseline levels until day 3 (Figure 3). Although there were no between-group differences on day 1, the recovery from a depressed neuroscore performance and locomotor hypoactivity was significantly better in the neurotropin group than in the control group (*p* < 0.05), while the exploratory ability (as represented by the central and total distance ratio) remained unaffected (Table S1).

**Figure 4:**
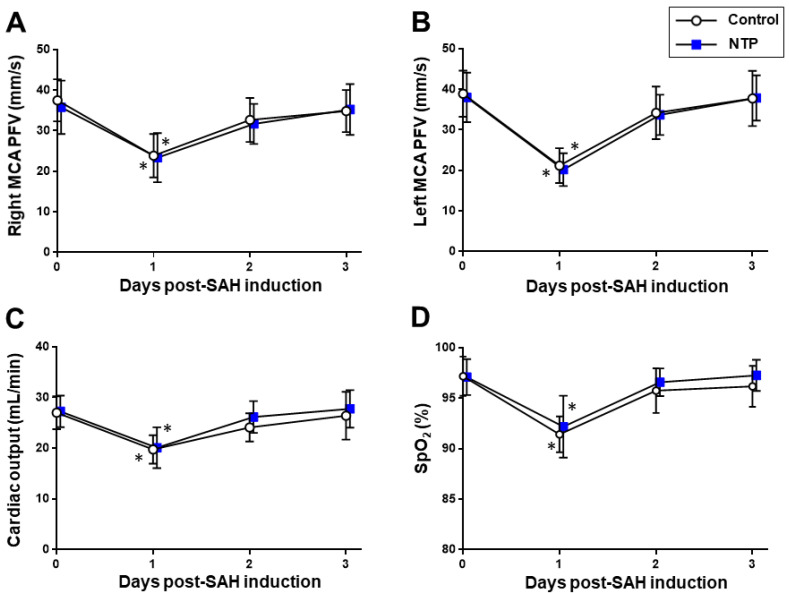
Time–course changes in the acute phase after SAH induction in the control and neurotropin groups. Peak systolic flow velocity (PFV) of the right (**A**) and left (**B**) middle cerebral arteries (MCA). Cardiac output (CO) (**C**). Oxygen saturation (SpO_2_) (**D**). * *p* < 0.05 vs. baseline; two-way ANOVA with a post hoc analysis (Bonferroni–Dunn’s test). NTP, neurotropin.

Meanwhile, there were no significant differences due to time–course changes in the body weight, SpO_2_, HR, CO, or peak systolic flow velocities of the middle cerebral arteries (*p* > 0.05) (Figure 4).

**Figure 5:**
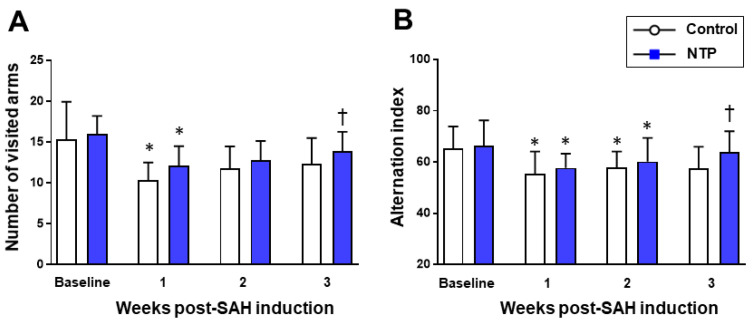
Time–course changes in the chronic phase after SAH induction in the control and neurotropin groups. Number of visited arms (**A**). Alternation index (**B**). * *p* < 0.05 vs. baseline; ^†^ *p* < 0.05 vs. control; two-way ANOVA with a post hoc analysis (Bonferroni–Dunn’s test). NTP, neurotropin.

The cognitive function assessment using a Y-maze test in the post-SAH chronic phase (Table S2) showed a significant reduction in both the number of visited arms and alternation index from the baseline levels (*p* <0.05) in the two groups. However, at week 3, the neurotropin group showed a significantly higher number of visited arms and higher alternation scores than the control group (*p* < 0.001) (Figure 5).

## 4. Discussion

There remains limited evidence on the neuroprotective effects of neurotropin on the central nervous system. This study found that neurotropin administered systemically early after SAH insult attenuated early brain infarction and improved cognitive impairments at the chronic phase. Further, it promoted recovery from acute neurological and functional impairments. To our best knowledge, this is the first animal experiment to demonstrate the neuroprotective effect of neurotropin for post-SAH treatment.

Neither creating SAH procedure nor the post-SAH administration of neurotropin systemically had any differences in the cardiorespiratory and cerebrovascular parameters from the control (Figure 2). This suggests that, although the neuroprotective effects of neurotropin are less likely to be a result of the hemodynamic changes via the systemic circulation, they could act preferentially in the central nervous system (CNS).

Apart from the well-known analgesic effects, the mechanisms underlying the CNS effect of neurotropin on post-SAH EBI have not been extensively investigated. Neurotropin facilitates the neurotrophin signaling pathway mediated by the tropomyosin-related kinase (Trk) receptor protein tyrosine kinase A, which is necessary for controlling the survival and differentiation of CNS neurons [31]. The existence of Trk that may trigger tumor resistance to rapamycin, an inhibitor of the mammalian target of rapamycin (mTOR), has been demonstrated in vivo [32], and the systemic application of rapamycin has achieved similar neuroprotective effects to the present data [19,21], supporting the importance of a signal transduction pathway. It has also been described that neurotropin alleviates the secondary damage after a spinal cord injury by inhibiting apoptosis, upregulating the neurotrophic factors, and downregulating proinflammatory cytokines, at least, in part, via the Janus kinase (JAK) signal transducer and activator of transcription (STAT) signaling to improve the functional performance [33]. We also reported the important role of the JAK–STAT3 pathway in the post-treatment neuroprotection of central rapamycin administration against post-SAH EBI [19], suggesting the possibility of some overlapping mechanisms. The anti-inflammatory properties of neurotropin could also contribute to the improvement of post-SAH EBI. A previous study showed that the intraperitoneal injection of neurotropin has neuroprotective effects against hypoxic–ischemic brain injury by reducing the cerebral infarct volume and mRNA expression of proinflammatory cytokines such as interleukin-6, tumor necrosis factor α, and interleukin-1β [12]. Furthermore, a novel platform for crosstalk between the neurotrophic and innate immune receptors showed that the neurotropin-treated cells also contained Toll-like receptor 4 [34], indicating the neuroprotective actions of neurotropin via the pleiotropic CNS pathways.

Notably, we found that neurotropin rescued the spatial memory impairment of mice in the chronic phase after SAH. It seems unlikely that the cognitive decline was influenced by the locomotive hypoactivity and/or neurological disturbance observed during the acute phase after SAH, because these parameters generally recovered close to the baseline levels around days 3–7 in our murine model of SAH [19,35]. Although the potential role of neurotropin in memory impairment has not been evaluated, the induction of BDNF either through its analgesic (descending pain inhibition) [36] or modulatory functions on neuroinflammation [37] may be involved. Recent studies of transgenic mice models of Alzheimer’s disease showed that neurotropin could alleviate oxidative stress, reduce neuroinflammation, and improve cognitive deficits possibly via the BDNF/NF-κB pathway [17,38,39], which may, in part, support a possible mechanism induced by the post-SAH cognitive decline.

This study had some limitations. First, this study did not mention age-related changes in brain tissue and systemic organ damages and neurocognitive responses to SAH similar to those seen in elderly patients, because young adult mice were used. Further investigations using murine models of brain aging and age-related neurodegenerative diseases, such as senescence-accelerated mouse strains [40], or old mice correlated with the human lifespan [41,42] may help to demonstrate those changes. Second, this study did not include sham-operated control mice to undergo the same procedure without vessel perforation. This lack of controls was partially offset by pre-procedure baseline measures in both groups, but was still a weakness of the approach. Thirdly, neurotropin should ideally be administered at the early post-SAH period, but the optimal timing and treatment protocol (e.g., dose and administration route) need to be validated based on the pharmacological profiles. The use of an endovascular perforation model may not be enough to effectively detect multidomain neurobehavioral deficits in C57BL/6 mice [35], although the severity of SAH was similar to those of humans. To understand the clinically relevant situations in patients with SAH, more specific experimental settings are required.

## 5. Conclusions

In conclusion, our translational results supported that neurotropin may be a novel potential drug for attenuating post-SAH EBI and the related motor and cognitive impairments. This effect seems to be a CNS neuroprotection independent of the systemic circulation. The clinical and prognostic values of neurotropin in response to SAH injury may be incorporated with our postoperative management protocol [43] for future directions.

## Data Availability

No big data repositories were needed. The raw data supporting the findings of this manuscript will be made available by the corresponding author (Tatsushi Mutoh) to any qualified researchers upon reasonable request.

## Supplementary Materials

The following are available online at https://www.mdpi.com/article/10.3390/biomedicines9060664/s1, Figure S1: Multimodal in vivo methods using cerebrovascular and brain imaging in mice (**A**). Representative MRI images of SAH grading by hypointensity signals on T2*WI (**B**) and post-SAH acute cerebral infarction (red arrows) detected by hyperintensity signals on DWI and T2WI (**C**). Figure S2: Representative images of flow velocities of the left middle cerebral artery (**A**) and left ventricular outflow tract (**B**) in mice before (baseline), immediately after SAH, and 24 h (day 1) after experimental SAH in a mouse treated with neurotropin after SAH induction. Table S1: Changes in the hemodynamic and functional parameters in the acute phase after murine SAH. Table S2: Changes in cognitive functions in the subacute to chronic phases after murine SAH.
