# Correction: Mutoh et al. Post-Event Application of Neurotropin Protects against Ischemic Insult toward Better Outcomes in a Murine Model of Subarachnoid Hemorrhage. *Biomedicines* 2021, *9*, 664

**DOI:** 10.3390/biomedicines12092033

**Published:** 2024-09-06

**Authors:** Tatsushi Mutoh, Shuzo Yamamoto, Takahiro Moriya

**Affiliations:** 1 Department of Aging Research and Geriatric Medicine, Institute of Development, Aging and Cancer, Tohoku University, Aoba-ku, Sendai 980-8575, Japan; 2 Department of Pharmacology, School of Pharmaceutical Sciences, Ohu University, Koriyama, Fukushima 963-8611, Japan

**Error in Figure**

In the original publication [1], there was a mistake in Figure S2 as published. A representative recording of the transcranial Doppler flow velocity tracing in the right panel of (A) was incorrectly placed due to unexpected technical error of the offline data output system. The corrected Figure S2 appears below.

**Figure S2:**
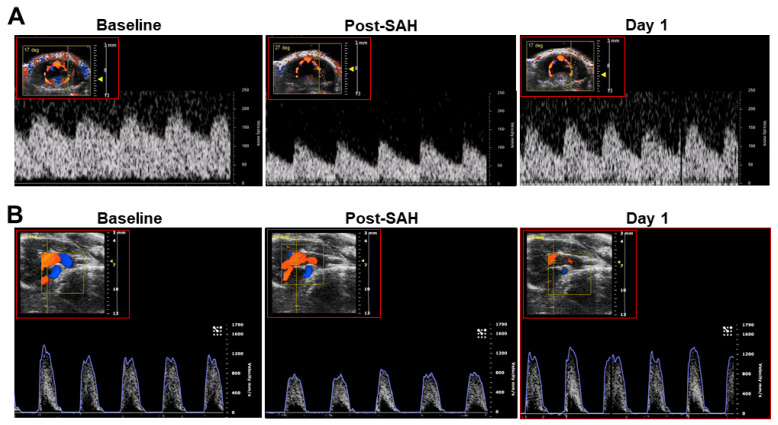
Representative images of flow velocities of the left middle cerebral artery (**A**) and left ventricular outflow tract (**B**) in mice before (baseline), immediately after SAH, and 24 h (day 1) after experimental SAH in a mouse treated with neurotropin after SAH induction.

**Updated Affiliation**

In the original publication [1], the affiliations listed for the authors Tatsushi Mutoh and Shuzo Yamamoto were the numbers 1 and 2. Affiliation 1 was: Department of Geriatric Medicine and Neuroimaging, Tohoku University Hospital, Aoba-ku, Sendai 980-8575, Japan; and Affiliation 2 was: Department of Aging Research and Geriatric Medicine, Institute of Development, Aging and Cancer, Tohoku University, Aoba-ku, Sendai 980-8575, Japan. These departments have merged into a single affiliation (Affiliation 1) and are no longer separate units. In addition, only the corresponding author’s email address needs to be displayed.

The authors state that the scientific conclusions are unaffected. This correction was approved by the Academic Editor. The original publication has also been updated.
