# Supplementary material for: Post-Event Application of Neurotropin Protects against Ischemic Insult toward Better Outcomes in a Murine Model of Subarachnoid Hemorrhage
Source: Biomedicines. 2021 Jun 10;9(6):664. doi: 10.3390/biomedicines9060664 (PMC8227975; doi:10.3390/biomedicines9060664)
Supplement: Supplementary file 1 [file biomedicines-09-00664-s001.zip › biomedicines-1232142-supplementary/biomedicines-1232142-supplementary.pdf]

biomedicines

MDPI

Article

Post-Event Application of Neurotropin Protects Against Ischemic Insult Toward Better Outcomes in a Murine Model of Subarachnoid Hemorrhage

Tatsushi Mutoh, Shuzo Yamamoto and Takahiro Moriya

Supplementary Materials

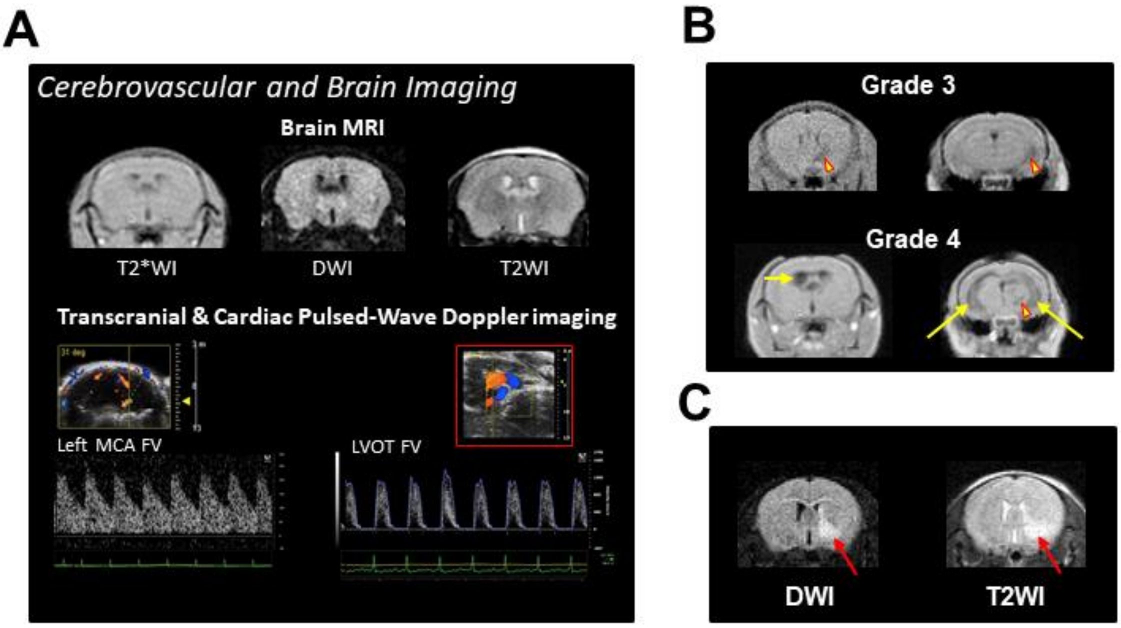

Figure S1. Multimodal in vivo methods using cerebrovascular and brain imaging in mice (**A**). Representative MRI images of SAH grading by hypointensity signals on T2*WI (**B**) and post-SAH acute cerebral infarction (red arrows) detected by hyperintensity signals on DWI and T2WI (**C**). Yellow arrows and arrow heads in (**B**) indicate intraventricular hemorrhage and SAH, respectively. MCA, middle cerebral artery; FV, flow velocity; LVOT, left ventricular outflow tract.

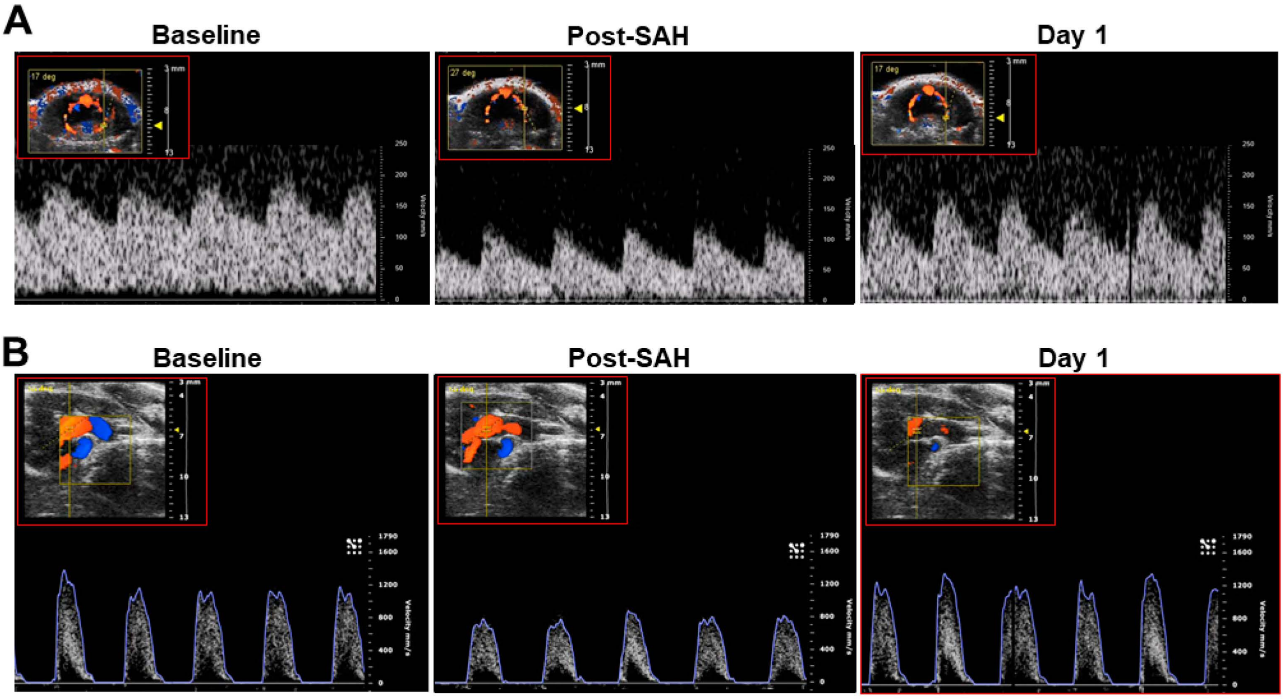

Figure S2. Representative images of flow velocities of the left middle cerebral artery (**A**) and left ventricular outflow tract (**B**) in mice before (baseline), immediately after SAH, and 24 h (day 1) after experimental SAH in a mouse treated with neurotropin after SAH induction.

Table S1. Changes in hemodynamic and functional parameters in acute phase after murine SAH.

	**Baseline**	**Day 1**	**Day 2**	**Day 3**	**Intergroup *p* value**
Body weight (g)					
Control	25.5 ± 1.3	23.2 ± 2.1*	24.0 ± 1.8	24.3 ± 1.5	0.368
NTP	25.3 ± 1.2	22.9 ± 1.7*	24.3 ± 1.7	25.0 ± 1.4	
Left MCA PFV					
Control	38.9 ± 5.7	21.2 ± 4.3*	34.2 ± 6.5	37.8 ± 6.8	0.946
NTP	38.0 ± 6.1	20.2 ± 4.0*	33.7 ± 5.0	37.8 ± 5.6	
Right MCA PFV					
Control	37.5 ± 5.2	23.8 ± 5.4*	32.7 ± 5.4	34.8 ± 5.2	0.784
NTP	35.8 ± 6.6	23.3 ± 6.1*	31.7 ± 4.9	35.3 ± 6.3	
Heart rate (min^{-1})					
Control	464 ± 41	427 ± 36	456 ± 37	455 ± 39	0.663
NTP	471 ± 40	425 ± 30	459 ± 34	460 ± 34	
Cardiac output (mL/min)					
Control	27.0 ± 3.2	19.8 ± 2.8*	24.1 ± 2.8	26.4 ± 4.7	0.005
NTP	27.3 ± 3.1	20.1 ± 4.0*	26.2 ± 3.2	27.8 ± 3.7	
SpO_2 (%)					
Control	97 ± 2	91 ± 2*	96 ± 2	96 ± 2	0.656
NTP	97 ± 2	92 ± 3*	97 ± 1	97 ± 2	
Neuroscore					
Control	18.0 ± 0.0	11.8 ± 1.7*	14.8 ± 1.9*	16.1 ± 1.4	0.026
NTP	18.0 ± 0.0	12.1 ± 2.4*	16.7 ± 1.4†	16.9 ± 1.2†	
Total distance (m/10 min)					
Control	53.3 ± 6.6	31.9 ± 6.7*	41.7 ± 5.9*	48.6 ± 5.4	0.038
NTP	56.6 ± 8.9	34.9 ± 6.5*	47.8 ± 6.0†	53.9 ± 6.5†	

Ratio of central/total distance					
Control	1.8 ± 0.5	1.4 ± 0.4	1.6 ± 0.5	1.6 ± 0.4	0.874
NTP	1.7 ± 0.4	1.5 ± 0.6	1.6 ± 0.3	1.7 ± 0.5	

Values are expressed as mean ± SD. $^{*}p < 0.05$ vs. Baseline; †$p < 0.05$ vs. Control. Two-way ANOVA with Bonferroni-Dunn correction for multiple comparisons. Within group p values for each variable were less than 0.0001 with the exception for the ratio of central/total distance ($p > 0.05$). MCA, middle cerebral artery; PFV, peak flow velocity; NTP, neurotropin.

Table S2. Changes in cognitive functions in subacute to chronic phase after murine SAH.

	**Baseline**	**Day 1**	**Day 2**	**Day 3**	**Intergroup *p* value**
Number of visited arms					
Control	6.3 ± 1.2	6.3 ± 1.2*	6.3 ± 1.2	6.3 ± 1.2	0.014
NTP	6.2 ± 1.3	6.2 ± 1.3*	6.2 ± 1.3	6.2 ± 1.3†	
Alternation index					
Control	65.1 ± 8.7	55.4 ± 8.7*	57.7 ± 6.3*	57.5 ± 8.4	0.028
NTP	66.2 ± 10.0	57.7 ± 5.6*	60.2 ± 9.2*	63.9 ± 8.1†	

Values are expressed as mean ± SD. $^{*}p < 0.05$ vs. Baseline; $^{\dagger}p < 0.05$ vs. Control. Two-way ANOVA with Bonferroni-Dunn correction for multiple comparisons. Within group p values for both parameters. NTP, neurotropin.
